# Native AMPA receptor architecture reveals SynDIG4 engagement and auxiliary subunit heterogeneity

**DOI:** 10.1126/sciadv.aee7973

**Published:** 2026-06-12

**Authors:** Chengli Fang, Eric Gouaux

**Affiliations:** ^1^ Vollum Institute, Oregon Health & Science University, Portland, OR 97239, USA.; ^2^ Howard Hughes Medical Institute, Oregon Health & Science University, Portland, OR 97239, USA.

## Abstract

AMPA-type glutamate receptors (AMPARs) are complex assemblies whose compositional heterogeneity underlies diverse excitatory signaling in the mammalian brain. Here, we determine high-resolution cryo–electron microscopy (cryo-EM) structures of native AMPAR complexes rapidly purified from mouse brain. These structures capture receptors in physiologically relevant assemblies containing distinct combinations of transmembrane AMPA receptor regulatory protein (TARP) and cornichon homolog (CNIH) auxiliary subunits and reveal unambiguous density for the brain-specific protein SynDIG4. The resolved topology and interaction network of SynDIG4 show that it engages the receptor through a CNIH-dependent interface and occupies a position adjacent to structural elements of GluA1 implicated in trafficking and synaptic plasticity. The diversity of auxiliary stoichiometries observed across native complexes highlights a flexible organizational scheme through which AMPARs incorporate distinct regulatory partners. These findings illuminate the organization of native AMPAR assemblies and define the structural context for SynDIG4 function in the mammalian brain.

## Teaser

SynDIG4 engages with GluA1 and CNIH on native AMPA receptor complexes to sculpt receptor phosphorylation and trafficking.

## INTRODUCTION

AMPA-type glutamate receptors (AMPARs) mediate fast excitatory synaptic transmission and play essential roles in brain development and synaptic plasticity (*1*). In the mammalian brain, these receptors do not function as isolated pore-forming tetramers. Instead, native AMPARs assemble with a remarkable constellation of auxiliary subunits, such as transmembrane AMPA receptor regulatory proteins (TARPs) and cornichon homologs (CNIHs), that fine-tune receptor trafficking, gating, and pharmacological properties (*1*–*3*). By modulating receptor abundance at synapses and adjusting channel opening in response to glutamate, these auxiliary proteins endow AMPARs with diverse functional profiles across neuronal types and circuits, thereby enhancing the diversity and adaptability of excitatory signaling in the central nervous system.

Multiple studies have defined how TARPs and CNIHs engage AMPARs and modulate their functional and structural properties (*4*–*6*). However, native AMPAR complexes in the brain exhibit heterogeneous compositions and how multiple auxiliary subunits assemble and collaboratively shape receptor architecture and function remains incompletely understood. A notable gap concerns SynDIG4, a brain-specific AMPAR-associated protein (also known as proline-rich transmembrane protein 1) implicated in synapse development, plasticity, and function (*7*). Previous native AMPAR studies reported only low-resolution peripheral density tentatively attributed to SynDIG4 (*5*), leaving its unambiguous identification, membrane topology, and interaction network unresolved. Thus, despite its functional importance, the structural role of SynDIG4 within native AMPAR assemblies has remained elusive.

To address these questions, we determined single-particle cryo–electron microscopy (cryo-EM) structures of native AMPAR complexes rapidly purified from mouse brain. These analyses reveal five major auxiliary subunit assemblies and yield high-resolution structures of SynDIG4-containing receptors, enabling definitive assignment of SynDIG4 and providing detailed structural insights into its mode of association and interactions with CNIH and the receptor transmembrane domain (TMD). Together, these findings expand our views of native AMPAR compositional diversity and illuminate how SynDIG4 contributes to auxiliary subunit organization in the brain.

## RESULTS

### Rapid isolation of native AMPAR complexes

**Fig. 1.**
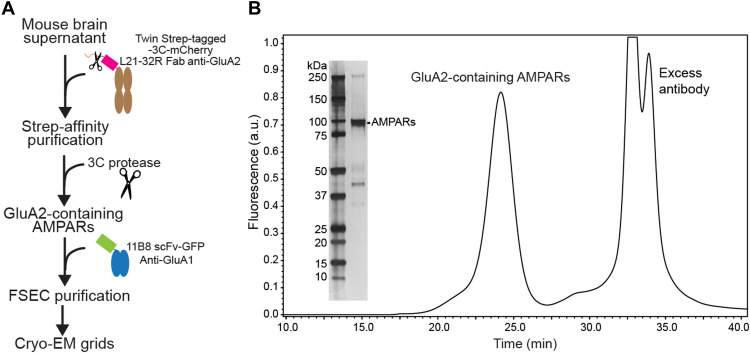
Isolation of GluA2-containing AMPARs from the whole brain. (**A**) Workflow for the purification of GluA2-containing AMPARs from mouse whole-brain tissue. (**B**) Representative FSEC profile of the native GluA2-containing AMPARs. The inset shows a silver-stained SDS-PAGE gel of the purified AMPAR complexes. a.u., arbitrary units.

To preserve the native subunit composition of AMPARs, we used a rapid immunoaffinity purification strategy to isolate receptors from mouse brain tissue (Fig. 1 and fig. S1) (*8*). Previous native purifications commonly relied on the GluA2 antibody 15F1, which recognizes an epitope within the amino-terminal domain (ATD) (*5*, *9*). Because ATD epitopes may coincide with binding sites for endogenous partners, we instead used a C-terminal domain (CTD) antibody. The engineered Fab (fragment antigen-binding) was fused to mCherry, enabling fluorescence-detection size-exclusion chromatography (FSEC) (*10*) to monitor AMPARs under low-abundance conditions (Fig. 1). The resulting FSEC profiles showed a sharp, monodisperse peak, and the purified sample exhibited high purity by SDS–polyacrylamide gel electrophoresis (SDS-PAGE). In addition, Western blotting and mass spectrometry analysis confirmed the presence of known AMPAR auxiliary and interacting proteins (fig. S1), consistent with preservation of native receptor assemblies. This workflow provided a sensitive quality control pipeline and enabled efficient isolation of native AMPAR complexes suitable for high-resolution cryo-EM analysis from as few as five mouse brains.

Despite using a CTD-directed antibody, no additional densities corresponding to potential extracellular partners were observed at the ATD layer of GluA2-containing AMPARs (fig. S2). Although mass spectrometry detected Noelin1 in the purified sample (fig. S1A), its absence in the cryo-EM maps suggests that its association with GluA2-containing receptors is either weak or highly dynamic. This interpretation aligns with previous findings that Noelin1 preferentially engages GluA4, rather than GluA2, when occupying the B and D subunits (*8*). In addition, these observations indicate that GluA2-containing AMPARs lack stable ATD-binding partners under our purification conditions. Consequently, our structural analysis focused on peripheral density features within the TMD layer, where auxiliary subunits are more likely to form stable and interpretable interactions with the receptor core.

### SynDIG4 association and positioning in AMPAR assemblies

**Fig. 2.**
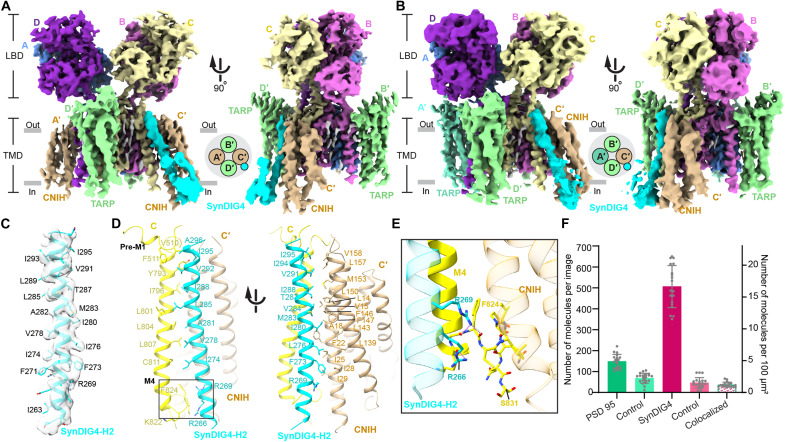
SynDIG4 interacts with CNIH and the M4 helix of AMPAR. (**A**) Cryo-EM map of the 2xTARP + 2xCNIH + SynDIG4 assembly showing SynDIG4 located at the interface between CNIH and the receptor M4 helix. (**B**) Cryo-EM map of the 3xTARP + 1xCNIH + SynDIG4 assembly revealing a similar SynDIG4 binding site. The insert shows the auxiliary subunit arrangements. TARPs, dark green and green; CNIHs, brown. SynDIG4 is shown in cyan. (**C**) Focused map of the SynDIG4 transmembrane region showing clear side-chain densities. (**D**) Detailed view of the interactions between SynDIG4, CNIH, and the receptor M4 helix. (**E**) Interactions of SynDIG4 with the M4 C-terminal loop, highlighting a potential stabilizing effect on this region. Possible hydrogen bonds are indicated by pink dashed lines. (**F**) SiMPull analysis showing that SynDIG4 does not colocalize with PSD95. Data represent mean ± SEM from *n* = 20 images. See Materials and Methods for details of control experiments.

The organization of AMPAR–auxiliary subunit complexes can be thought of as “onion like” where there is a central core of receptor subunits surrounded by an inner layer of auxiliary subunits that primarily modulate receptor function and an outer layer that largely participates in receptor trafficking and localization, thereby enabling the precise tuning of receptor activity and location (*11*, *12*). Inspection of the transmembrane periphery revealed a consistent, well-defined density adjacent to the CNIH layer in several three-dimensional (3D) classes. The location and overall shape of these density features corresponds to identification of an auxiliary subunit previously attributed to SynDIG4 in low-resolution native AMPAR maps (*5*), suggesting that our dataset captured this brain-specific regulatory protein. SynDIG4 plays a substantial role in excitatory synaptic plasticity, particularly within the hippocampus (*13*), and is crucial for learning and memory, likely by influencing AMPAR trafficking and surface expression (*7*, *14*–*16*). Focused 3D classification resolved a density consistent with the predicted architecture of SynDIG4, comprising a short membrane re-entrant helix (H1a-H1b) and a single canonical transmembrane helix (H2) (figs. S3 and S4, G to I) (*17*, *18*). On this basis, we identified two SynDIG4-containing receptor assemblies, 2xTARP + 2xCNIH + SynDIG4 and 3xTARP + 1xCNIH + SynDIG4, constituting 8 and 3% of the total particles, respectively (Fig. 2, Table 1, and figs. S2 to S4). Although these populations represent a minority of the total dataset, their clear structural features and consistent auxiliary architecture highlight SynDIG4 as a bona fide component of a subset of native AMPAR assemblies.

**Table 1.** Cryo-EM data collection, refinement, and validation statistics. FSC, Fourier shell correlation; RMS, root mean square.

	LBD-TMD, 4xTARP (EMD-74377) (PDB 9ZKM)	TMD, 4xTARP (EMD-74378) (PDB 9ZKN)	LBD-TMD, 2xTARP + 2xCNIH (EMD-74379) (PDB 9ZKO)	TMD, 2xTARP + 2xCNIH (EMD-74380) (PDB 9ZKP)
Data collection and processing				
Microscope	Titan Krios G4	Titan Krios G4	Titan Krios G4	Titan Krios G4
Detector	BioQuantumK3	BioQuantumK3	BioQuantumK3	BioQuantumK3
Magnification	105,000	105,000	105,000	105,000
Voltage (kV)	300	300	300	300
Electron exposure (e−/Å^2^)	50	50	50	50
Defocus range (μm)	−1.2 to −2.2	−1.2 to −2.2	−1.2 to −2.2	−1.2 to −2.2
Pixel size (Å)	0.825	0.825	0.825	0.825
No. of micrographs	20,208	20,208	20,208	20,208
Initial particle images (no.)	1,503,517	1,503,517	1,503,517	1,503,517
Final particle images (no.)	112,093	112,093	111,937	111,937
Symmetry imposed	C1	C1	C2	C2
Map resolution (Å)	3.13	2.80	3.04	2.87
FSC threshold (0.143)				
Map resolution range (Å)	2.62–51.19	2.50–46.09	1.77–41.76	2.53–38.67
Refinement				
Initial model used (PDB code)	7LEP	7LEP	7LEP	7LEP
Model resolution (Å)	3.1 (3.4)	2.8 (3.0)	3.0 (3.3)	2.8 (3.0)
FSC threshold	0.143 (0.5)	0.143 (0.5)	0.143 (0.5)	0.143 (0.5)
Map sharpening *B* factor (Å^2^)	−83.9	−81.6	−98.3	−90.3
Model composition				
Nonhydrogen atoms	17,822	10,100	17,195	9,502
Protein residues	2,316	1,253	2,215	1,139
Ligands	POV:8, PLM:12, OLC:8	POV:8, PLM:11, OLC:8	POV:8, PLM:12, OLC:8	POV:8, PLM:12, OLC:8
*B* factors (Å^2^)				
Protein	142.74	144.90	149.27	120.63
Ligand	148.93	153.42	144.55	135.90
RMS deviations				
Bond lengths (Å)	0.003	0.004	0.004	0.004
Bond angles (°)	0.737	0.734	0.745	0.682
Validation				
MolProbity score	1.69	1.45	1.55	1.27
Clashscore	6.32	6.59	6.75	5.08
Poor rotamers (%)	0.00	0.00	0.00	0.11
Ramachandran plot				
Favored (%)	95.03	97.59	96.96	98.16
Allowed (%)	4.97	2.41	3.04	1.84
Disallowed (%)	0.00	0.00	0.00	0.00
	LBD-TMD, 3xTARP + 1xCNIH (EMD-74381) (PDB 9ZKQ)	TMD, 3xTARP + 1xCNIH (EMD-74382) (PDB 9ZKR)	LBD-TMD, 3xTARP (EMD-74387) (PDB 9ZKW)	LBD-TMD, 2xTARP (EMD-74386) (PDB 9ZKV)
Data collection and processing				
Microscope	Titan Krios G4	Titan Krios G4	Titan Krios G4	Titan Krios G4
Detector	BioQuantumK3	BioQuantumK3	BioQuantumK3	BioQuantumK3
Magnification	105,000	105,000	105,000	105,000
Voltage (kV)	300	300	300	300
Electron exposure (e−/Å^2^)	50	50	50	50
Defocus range (μm)	−1.2 to −2.2	−1.2 to −2.2	−1.2 to −2.2	−1.2 to −2.2
Pixel size (Å)	0.825	0.825	0.825	0.825
No. of micrographs	20,208	20,208	20,208	20,208
Initial particle images (no.)	1,503,517	1,503,517	1,503,517	1,503,517
Final particle images (no.)	45,742	45,742	51,529	52,124
Symmetry imposed	C1	C1	C1	C1
Map resolution (Å)				
FSC threshold (0.143)	3.36	3.08	3.33	3.45
Map resolution range (Å)	2.91–56.56	2.73–49.88	2.88–50.82	1.75–56.56
Refinement				
Initial model used (PDB code)	7LEP	7LEP	7LEP	7LEP
Model resolution (Å)	3.3 (3.6)	3.1 (3.3)	3.3 (3.6)	3.4 (3.7)
FSC threshold	0.143 (0.5)	0.143 (0.5)	0.143 (0.5)	0.143 (0.5)
Map sharpening *B* factor (Å^2^)	−66.3	−74.0	−72.1	−78.5
Model composition				
Nonhydrogen atoms	17,595	9,928	16,169	14,900
Protein residues	2,286	1,203	2,130	1,968
Ligands	POV:6, PLM:10, OLC:8	POV:7, PLM:10, OLC:8	POV:5, PLM:6, OLC:8	POV:3, PLM:4, OLC:8
*B* factors (Å^2^)				
Protein	145.29	134.42	132.69	147.17
Ligand	145.26	142.14	140.71	154.70
RMS deviations				
Bond lengths (Å)	0.004	0.004	0.003	0.003
Bond angles (°)	0.791	0.809	0.698	0.701
Validation				
MolProbity score	1.68	1.46	1.60	1.59
Clashscore	6.89	6.20	5.65	5.68
Poor rotamers (%)	0.00	0.00	0.20	0.07
Ramachandran plot				
Favored (%)	95.72	97.39	95.75	95.97
Allowed (%)	4.28	2.61	4.25	4.03
Disallowed (%)	0.00	0.00	0.00	0.00
		LBD-TMD, 2xTARP + 2xCNIH + SynDIG4 (EMD-74383) (PDB 9ZKS)	TMD, 2xTARP + 2xCNIH + SynDIG4 (EMD-74384) (PDB 9ZKT)	LBD-TMD, 3xTARP + 1xCNIH + SynDIG4 (EMD-74385) (PDB 9ZKU)
Data collection and processing				
Microscope		Titan Krios G4	Titan Krios G4	Titan Krios G4
Detector		BioQuantumK3	BioQuantumK3	BioQuantumK3
Magnification		105,000	105,000	105,000
Voltage (kV)		300	300	300
Electron exposure (e−/Å^2^)		50	50	50
Defocus range (μm)		−1.2 to −2.2	−1.2 to −2.2	−1.2 to −2.2
Pixel size (Å)		0.825	0.825	0.825
No. of micrographs		20,208	20,208	20,208
Initial particle images (no.)		1,503,517	1,503,517	1,503,517
Final particle images (no.)		38,978	38,978	17,066
Symmetry imposed		C1	C1	C1
Map resolution (Å) FSC threshold (0.143)		3.36	3.04	3.75
Map resolution range (Å)		1.76–50.70	2.80–51.17	3.17–59.09
Refinement				
Initial model used (PDB code)		7LEP	7LEP	7LEP
Model resolution (Å)		3.3 (3.7)	3.0 (3.4)	3.7 (4.1)
FSC threshold		0.143 (0.5)	0.143 (0.5)	0.143 (0.5)
Map sharpening *B* factor (Å^2^)		−63.5	−74.0	−47.0
Model composition				
Nonhydrogen atoms		16,983	9,580	17,486
Protein residues		2,267	1,201	2,317
Ligands		POV:3, PLM:6, OLC:8	POV:3, PLM:6, OLC:9	POV:1, PLM:4, OLC:7
*B* factors (Å^2^)				
Protein		168.09	150.21	165.78
Ligand		142.70	149.19	135.34
RMS deviations				
Bond lengths (Å)		0.004	0.004	0.004
Bond angles (°)		0.905	0.749	0.862
Validation				
MolProbity score		1.71	1.32	1.62
Clashscore		6.31	4.98	6.67
Poor rotamers (%)		0.00	0.00	0.06
Ramachandran plot				
Favored (%)		94.78	97.73	96.21
Allowed (%)		5.22	2.27	3.79
Disallowed (%)		0.00	0.00	0.00

SynDIG4 interacts with CNIH and the M4 helix of the receptor in both assemblies (Fig. 2), forming a prominent auxiliary subunit to auxiliary subunit interface that is primarily mediated through its contact with CNIH. Notably, only one SynDIG4 is bound in the 2xTARP + 2xCNIH + SynDIG4 assembly, which differs from previous low-resolution maps where two SynDIG4 molecules were observed, each associating with one of the two CNIH subunits (*5*). This discrepancy could arise from asymmetric SynDIG4 occupancy across native AMPAR complexes, which may have been averaged into an apparent twofold symmetry in previous reconstructions. Alternatively, it may reflect partial loss of weakly associated SynDIG4 during purification or the coexistence of multiple heterogeneous complexes in vivo. Future experiments are required to resolve these different scenarios.

Focused refinement improved the local resolution, enabling confident side-chain modeling and unambiguous placement of SynDIG4 (Fig. 2C). In this conformation, the H2 of SynDIG4 contacts both the pre-M1 and M4 helices of GluA1, a subunit whose activity-dependent trafficking and synaptic recruitment play a central role in long-term potentiation (Fig. 2D) (*19*). Notably, SynDIG4 binding does not visibly alter the overall conformation of the ligand-binding domain–transmembrane domain (LBD-TMD), CNIH, or the M4 helix (fig. S5, I and J), indicating that its incorporation is compatible with maintaining the core receptor architecture. Instead, SynDIG4 occupies a peripheral site adjacent to regulatory elements of GluA1, a location that may permit modest stabilization of intersubunit contacts or subtly influence how GluA1-containing receptors are positioned within the membrane environment, although additional studies will be required to establish any functional consequences.

### SynDIG4 modulation of GluA1 and synaptic distribution

Previous work reported that loss of SynDIG4 enhances phosphorylation of GluA1 at S831, a modification known to strongly influence receptor trafficking (*15*, *19*), suggesting that SynDIG4 normally acts to limit phosphorylation at this site. In our high-resolution structure, SynDIG4 stabilizes the M4 helix of receptor subunits at the A or C positions, which enabled modeling additional C-terminal residues downstream of M4 (Fig. 2E and fig. S4J). The resulting model places the loop harboring S831 in proximity to R266 of SynDIG4 and the M1 helix of CNIH, revealing a potential local interaction network surrounding this regulatory loop. This configuration raises the possibility that SynDIG4 helps organize the S831-containing loop in a conformation that may partially restrict kinase access and thereby modulate phosphorylation at this site. Such an arrangement is consistent with earlier observations linking SynDIG4 loss to elevated S831 phosphorylation, although the structural data alone cannot establish causality. Native SynDIG4-containing AMPARs are compositionally heterogeneous, making it challenging to reconstitute complete and homogeneous complexes and to perform functional assays on these defined receptor complexes. Therefore, we have focused our efforts on structural characterization of receptors from the native brain, leaving functional validation to future studies.

To understand whether the SynDIG4 receptor complex is localized to synapses, we asked whether we could detect colocalization of SynDIG4 with postsynaptic density protein 95 (PSD95). However, single-molecule pull-down (SiMPull) (*20*) experiments revealed that SynDIG4 does not colocalize with PSD95 (Fig. 2F), consistent with previous reports (*13*), although this observation could, in part, be the consequence of dissociation of PSD95 and TARP interactions by digitonin. This suggests that SynDIG4-containing AMPARs are likely excluded from PSD95-enriched postsynaptic densities, indicating a potential role for SynDIG4 in regulating the trafficking receptors but not their localization in the postsynaptic density. In line with this idea, recent work has proposed that SynDIG4 may associate preferentially with AMPARs residing in endosomal compartments rather than with receptors anchored at mature synapses (*16*). Under this model, SynDIG4 could act to transiently retain AMPARs within endosomes and release them upon activity-dependent stimuli, providing a mechanistic explanation for why SynDIG4-containing receptors are rarely detected within PSD95-marked postsynaptic densities.

### Stoichiometric diversity of TARPs and CNIHs

**Fig. 3.**
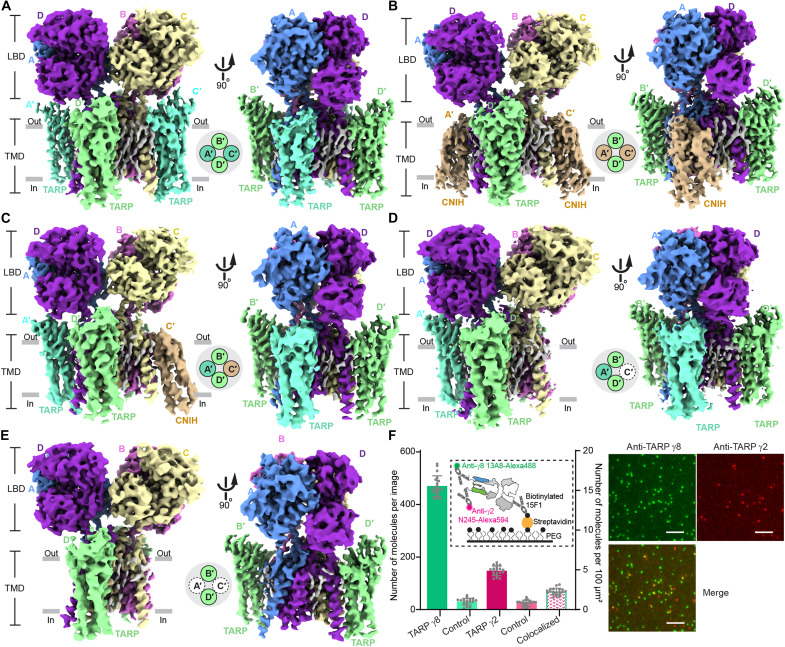
Cryo-EM reveals distinct auxiliary subunit stoichiometries and arrangements in native AMPAR complexes. (**A** to **E**) Representative cryo-EM maps of LBD-TMD mix assemblies exhibiting diverse auxiliary subunit compositions, including receptors with four TARPs (A′, B′, C′, and D′), mixed assemblies containing TARPs (B′ and D′), and CNIHs (A′ and C′) or those with varying numbers and configurations of TARPs and CNIHs. The insert shows the auxiliary subunit arrangements. TARPs are in dark green and light green. CNIHs are brown. A white circle represents no auxiliary subunit. (**F**) SiMPull analysis shows that GluA2-containing AMPARs copurify more frequently with TARP γ8 than with γ2 in whole-brain extracts and that γ2 and γ8 can coexist within the same receptor complex. Insert: schematic of the SiMPull assay. PEG, polyethylene glycol. Data represent mean ± SEM from *n* = 20 images. Scale bars, 5 μm. Representative images from three replicate experiments are shown. See Materials and Methods for details of control experiments.

Our cryo-EM results also yielded high-resolution maps that allowed unambiguous assignment of three symmetric auxiliary subunit assemblies: 4xTARP, 2xTARP + 2xCNIH, and 2xTARP. Notably, two assemblies displayed asymmetric arrangements, including 3xTARP + 1xCNIH and 3xTARP. These complexes were reconstructed at 3.13-, 3.04-, 3.45-, 3.36-, and 3.33-Å resolutions for the LBD-TMD layers, representing 23, 31, 10, 13, and 23% of the total particle population, respectively (Fig. 3, Table 1, and figs. S2 to S4), highlighting the structural heterogeneity and variable stoichiometry of native AMPAR complexes. Each complex displayed the canonical tetrameric AMPAR architecture, with approximate fourfold symmetry in the TMD and twofold symmetry in the LBD. Despite their overall similarity, peripheral densities within the TMD revealed distinct patterns of auxiliary subunit organization. In all complexes, TARPs consistently occupy the B′ and D′ positions, whereas the A′ and C′ positions display greater variability in auxiliary subunit composition (fig. S2E), reminiscent of the organization observed in native calcium-permeable (CP)-AMPAR assemblies (*8*). The absence of a 4xCNIH assembly, which has been observed in recombinant GluA2-CNIH3 preparations (*21*), suggests that such stoichiometries may be disfavored or less stable in the native brain environment. Although a previous report shows there exist partially TARP-bound receptors (*22*), it remains unclear whether the 3xTARP and 2xTARP assemblies in our results represent physiological species or result from subunit dissociation during purification.

AMPAR gating efficacy and kinetics are strongly influenced by the composition and stoichiometry of associated auxiliary subunits (*22*, *23*). To gain structural insights into how auxiliary subunits shape receptor activation, we next compared the three assemblies in which the A′B′C′D′ positions are fully occupied by TARPs or by combinations of TARPs and CNIHs (fig. S5). The conformation of each LBD dimer remained similar across assemblies, but clear rearrangements were observed between LBD dimers. The 4xTARP and 3xTARP + 1xCNIH complexes adopt a more compact dimer-dimer interface than the 2xTARP + 2xCNIH assembly, whereas the overall TMD architecture remains preserved (fig. S5). Consistent with these structural differences, previous electrophysiological studies have shown that TARPs modulate AMPAR gating in a stoichiometry-dependent manner. Specifically, increasing TARP occupancy generally enhances the overall extent of receptor modulation, although its effects on kinetic parameters are not uniform. For instance, assemblies with two TARPs exhibit faster activation, deactivation, and desensitization than fully TARP-occupied receptors (*1*, *22*–*24*). Beyond stoichiometry, TARP-dependent modulation is further sculpted by subtype-specific properties, as type 1a and type 1b TARPs differ in their extracellular loop composition and functional impact (*1*, *25*). Therefore, the structural changes observed here may reflect not only differences in TARP stoichiometry but also subtype-specific interactions. Furthermore, CNIH-associated receptors display slower deactivation and desensitization (*1*, *23*, *26*), a feature that may relate to the more “relaxed” LBD arrangement observed in the 2xTARP + 2xCNIH complex. Together, these observations suggest that auxiliary subunit-dependent differences in LBD geometry, governed by TARP stoichiometry and subtype as well as CNIH incorporation, contribute to distinct modes of AMPAR gating modulation.

3D classification of the ATD layer revealed that auxiliary subunit assembly does not depend on the specific AMPAR subunit composition. Across all complexes, the major assembly patterns were observed in similar proportions regardless of receptor subtype (fig. S2D). These results suggest that the principal modes of auxiliary subunit assembly are conserved across different AMPAR subtypes, indicating a lack of strict subunit selectivity in auxiliary subunit association. Nevertheless, the expression and assembly of auxiliary subunits exhibit neuronal cell type–specific patterns (*1*, *27*).

### TARP γ2/γ8 coexistence in native AMPARs

TARPs γ2 and γ8 are two of the most functionally influential AMPAR auxiliary subunits, and although both are broadly expressed, they show distinct regional expression biases, γ2 is enriched in cerebellum, whereas γ8 is more abundant in hippocampus and cortex. These patterns raise the question of whether native AMPARs assemble with a single predominant TARP subtype or whether multiple subtypes can coexist within the same receptor complex. To address this, we examined subtype incorporation using the SiMPull experiment. GluA2-containing AMPARs from whole-brain extracts copurified more frequently with γ8 than with γ2 (Fig. 3F), consistent with the predominance of γ8 in forebrain regions (*12*). Colocalization analysis revealed that γ2 and γ8 can coexist within the same receptor complex, demonstrating that native AMPAR assemblies are not limited to a single TARP subtype. Instead, they can incorporate multiple TARP family members simultaneously, forming mixed-subtype auxiliary architectures. This mixed incorporation adds compositional heterogeneity to the auxiliary shell surrounding AMPARs, expanding the possible mechanisms for tuning receptor behavior across circuits. However, in our current cryo-EM reconstructions, γ2 and γ8 densities are highly similar, and further classification does not allow unambiguous assignment of these subtypes within individual complexes. Future studies using complementary approaches, such as targeted labeling or specific ligands, may help to resolve the composition and functional consequences of mixed-TARP AMPAR complexes.

## DISCUSSION

Our study provides a structural framework for understanding how SynDIG4 associates with native AMPAR assemblies and contributes to their compositional and regulatory diversity in the mammalian brain. By rapidly purifying receptors from mouse brain tissue, we captured AMPAR complexes in assemblies that more faithfully reflect their physiological environments compared to recombinant systems. This approach enabled us to resolve SynDIG4 as a previously ambiguous auxiliary component and to delineate its interactions within heterogeneous AMPAR architectures.

One notable implication of our structural analysis is that SynDIG4 occupies a peripheral position near the GluA1 pre-M1/M4 helices and the S831 phosphorylation loop—an arrangement that offers a structural context for interpreting prior observations connecting SynDIG4 to GluA1-dependent plasticity. Although the present data do not define functional effects directly, the geometric relationship between SynDIG4, CNIH, and the GluA1 regulatory loop raises the possibility that SynDIG4 contributes to shaping the local environment that governs kinase accessibility or receptor stabilization. This arrangement outlines a structurally grounded model that could explain the reported SynDIG4 effects on S831 phosphorylation and GluA1 trafficking and motivates future studies aimed at testing these mechanistic possibilities.

Together, our findings indicate that SynDIG4 operates within multiauxiliary AMPAR assemblies rather than as an independent regulatory factor. The structures reveal that native AMPAR organization arises from combinatorial auxiliary configurations, in which different accessory proteins coexist and collectively sculpt receptor architecture. Within this framework, SynDIG4 assembles alongside TARPs and CNIHs, without perceptibly influencing receptor structure, thus suggesting that SynDIG4 may be more important for receptor trafficking than in modulating receptor function. Such combinatorial organization suggests that AMPAR regulation in the brain reflects the balance of multiple auxiliary components, providing a structural basis for tuning receptor localization and properties across synapses and developmental stages.

More broadly, native AMPARs display extensive auxiliary subunit diversity, populating multiple stoichiometries, including 4xTARP, 2xTARP + 2xCNIH, and 3xTARP + 1xCNIH assemblies, highlighting a compositional flexibility that enables fine-tuning of receptor properties in the brain. Although the functional implications of this diversity remain to be fully defined, the range of assemblies observed here suggests that AMPAR auxiliary organization does not follow a single dominant pattern across neuronal populations. Future studies combining electrophysiology with in situ structural approaches will be important for clarifying how TARPs, CNIHs, and SynDIG4 coordinate AMPAR regulation in physiological contexts.

## MATERIALS AND METHODS

### Purification of anti-GluA1 11B8 scFv-GFP

Expression and purification of the 11B8 scFv (single-chain variable fragment) were performed as previously described (*8*).

### Purification of the Twin-Strep–tagged L21-32R Fab-mCherry

Expression and purification of the L21-32R Fab were performed as previously described (*8*).

### Purification of GluA2-containing AMPARs from whole brain

GluA2-containing AMPARs were purified from whole mouse brains following previously established protocols (*8*). Mouse whole brains were gently homogenized and solubilized. The clarified supernatant was incubated with an excess amount of L21-32R Fab-mCherry-3C-Twin-Strep II for 10 min at 4°C to allow complex formation. The mixture was then applied to a gravity column packed with Strep-Tactin XT resin. After binding, the resin was washed, and the protein complex was eluted by incubating with one column volume of 3C protease (0.5 mg/ml) in FSEC buffer for 10 min at 4°C. The eluted sample was subsequently incubated with an excess of 11B8 scFv and concentrated. Final purification was performed by FSEC, and the peak fractions were pooled and concentrated using a 100-kDa molecular weight cutoff concentrator.

### SiMPull assay

Mouse brains were gently homogenized in homogenization buffer [20 mM tris-HCl (pH 8.0), 150 mM NaCl, 0.8 μM aprotinin, leupeptin (2 μg/ml), and 2 μM pepstatin A] using a Dounce homogenizer at a ratio of 3 ml of buffer per brain. Digitonin was then added to a final concentration of 2% (w/v), and the mixture was incubated at 4°C for 30 min with gentle agitation to solubilize membrane proteins. The lysate was clarified by ultracentrifugation at 200,000*g* for 20 min at 4°C, and the supernatant was collected for downstream assays. SiMPull measurements were carried out in assay buffer [20 mM tris-HCl (pH 8.0), 150 mM NaCl, 0.075% (w/v) digitonin, and bovine serum albumin (0.2 mg/ml)]. Passivated and biotinylated total internal reflection fluorescence (TIRF) flow chambers were prepared as previously described (*5*). Each chamber was incubated with 10 μl of streptavidin (0.25 mg/ml) for 5 min, followed by a wash with 30 μl of assay buffer. Biotinylated anti-GluA2 capture antibody [15F1 monoclonal antibody (mAb)-biotin, 60 nM] was then applied for 5 to 10 min. Negative control chambers lacking the capture antibody were processed in parallel. After washing, 30 μl of the solubilized brain extract diluted 1:100 in assay buffer was introduced and incubated for 5 to 10 min. Following another wash, fluorescently labeled detection antibodies for TARP γ8 (13A8 mAb–Alexa Fluor 488) and TARP γ2 (N245/1R mAb–Alexa Fluor 594) were added at 30 nM and incubated for 10 min. Chambers were washed and then imaged using a Leica DMi8 TIRF microscope equipped with a 100× oil-immersion objective and an Andor iXon Ultra 888 electron-multiplying charge-coupled device (EMCCD) camera, as described previously (*5*), with a 133–by–133 μm imaging area and a 130-nm pixel size.

For SynDIG4 and PSD95 colocalization analysis, brains from *PSD95-EGFP* knock-in mice (in which EGFP is fused to the C terminus of PSD95 in a C57BL/6 background) (*28*) were homogenized using the same method. Biotinylated anti-GluA1 capture antibody (11B8 mAb-biotin, 60 nM) was applied for 5 to 10 min, and negative control chambers were processed in parallel. After washing, solubilized brain extract (1:100 dilution) was incubated for 5 to 10 min, followed by the addition of fluorescently labeled anti-SynDIG4 antibody (L102/45 mAb–Alexa Fluor 555, 30 nM) for 10 min. Chambers were washed and then imaged.

For colocalization analysis, images from two color channels were acquired sequentially within the same region of interest under different wavelengths, and the position of each molecule was calculated using ComDet in ImageJ (FIJI). Molecule positions were determined with a custom Python script (*5*). Molecules located within a five-pixel were classified as colocalized. At least 20 images were averaged for each experiment. Data were analyzed with GraphPad Prism.

### Cryo-EM sample preparation and data acquisition

Cryo-EM grids were prepared by applying 3 μl of freshly purified native AMPAR (0.1 mg/ml) onto Quantifoil R2/1 200-mesh gold grids coated with a 2-nm continuous carbon layer. The grids were glow-discharged in the presence of amylamine for 30 s at 15 mA. Following a 10-s incubation, excess liquid was blotted away for 3 s (blot force 0), and the grids were rapidly vitrified in liquid ethane using a Vitrobot maintained at 16°C and 100% humidity. Data were collected on a Titan Krios microscope equipped with a Gatan K3 direct electron detector operating in super-resolution mode (physical pixel size of 0.825 Å) and an energy filter with a 20-eV slit width. Images were acquired at a defocus range of −1.2 to −2.2 μm, using an exposure rate of around 15 e^−^ per pixel per second over 45 frames, corresponding to a total exposure time of 2.25 s resulting in a total dose of 50 e^−^ Å^−2^.

### Cryo-EM data processing

Cryo-EM data were processed using cryoSPARC (v4.7) (*29*). Beam-induced motion was corrected using patch motion correction, and contrast transfer function (CTF) parameters were estimated by patch CTF estimation. Particles were initially picked using the Blob Picker to generate 2D class averages displaying clear receptor features, which were then used as templates for Template Picker. From 20,208 micrographs, particles from both blob- and template-based picking were extracted with a 512 × 512 pixel box (4× binned) and subjected to 2D classification, with only ice feature classes discarded. Ab initio reconstruction (*N* = 6) and four rounds of heterogeneous refinement were performed to remove false positives. Combining these two particle sets yielded 1,503,517 particles after removing duplicates. The particles were then reextracted at full resolution (512 × 512 pixels, bin 1). After nonuniform refinement, signals from the ATD layer were subtracted, and the box size was reduced to 392 × 392 pixels and then down-sampled by 2× binning. Two rounds of ab initio and heterogeneous refinement (*N* = 4) produced 912,443 particles with well-defined receptor features, of which 532,765 showed clear auxiliary subunit densities. A 3D classification (*N* = 4, without alignment) focused on the A′C′ positions revealed distinct auxiliary subunit compositions, which were used as references for subsequent heterogeneous refinement. Another round of classification focusing on the TMD and B′D′ positions yielded 798,156 particles exhibiting continuous transmembrane helical densities. Heterogeneous refinement (*N* = 4; initial resolution = 6 Å) of this subset produced four major classes with 183,022, 251,410, 190,412, and 173,312 particles, corresponding to distinct auxiliary subunit arrangements. The class with 190,412 particles exhibited low TMD resolution. Further classification and refinement did not improve the map, and high-resolution structural interpretation was not possible; therefore, this class was not subjected to additional processing.

Particles from the first two classes (434,432 in total) were combined and subjected to ab initio and heterogeneous refinement (*N* = 4), yielding 333,754 high-quality particles. 3D classification (*N* = 3) without alignment focusing on C′ position resolved 4xTARP, 3xTARP, and 3xTARP + 1xCNIH assemblies, comprising 112,093, 111,910, and 109,751 particles, respectively. (i) For 4xTARP assembly, 112,093 particles subjected to nonuniform refinement yielded a 3.13-Å map (C1, bin 1) by the gold standard Fourier shell correlation (0.143). This map shows 4 auxiliary subunit densities around the TMD at A′B′C′D′ with prominent extracellular protrusions. Further local refinement focused on TMD layer reached 2.8-Å resolution. (ii) For 3xTARP assembly, further classification of the 111,910 particles identified 3xTARP (51,529 particles) and 2xTARP (52,124 particles) states, refined to 3.33- and 3.45-Å LBD-TMD maps, respectively. (iii) For 3xTARP + 1xCNIH, heterogeneous refinement (*N* = 4) of 109,751 particles yielded a class (50,124 particles) showing weak SynDIG4 signals around CNIH. Focused classification on the C′ position identified a 3xTARP + 1xCNIH + SynDIG4 state (17,066 particles), refined to 3.75 Å (C1, bin 1). One round of 3D classification (*N* = 2) without alignment focusing on A′ position was then performed on the rest of the particles (92,685 particles) from the 109,751 particles set, resulting in one class (45,742 particles) with clear CNIH signals, and then subjected to nonuniform refinement yielding a 3.36-Å map (C1, bin 1), designated 3xTARP + 1xCNIH assembly. Further local refinement focused on a TMD layer was performed, resulting in a 3.08-Å map.

The fourth major class (173,312 particles) contained a subset (38,978 particles) displaying clear SynDIG4 density, refined to a 3.36-Å map (C1, bin 1) and further improved to 3.04 Å (TMD focused) and 3.21 Å (SynDIG4-CNIH focused). The remaining 134,334 particles yielded a 2xTARP + 2xCNIH assembly (111,937 particles) refined to 3.04 Å (C2, bin 1) and 2.87 Å after local refinement focused on TMD layer, showing four auxiliary subunits surrounding the TMD, TARPs at the B′D′ positions with prominent extracellular protrusions and CNIHs at the A′C′ positions.

### Model building

The structural modeling was carried out using rigid body fitting of the structure of LBD-TMD [Protein Data Bank (PDB): 7LEP] and AlphaFold2-predicted models from the Alpha Fold DB using UCSF Chimera (*5*, *17*, *30*). The structure was manually adjusted in Coot, with stereochemical restraints applied (*31*) and further refined by real-space refinement using Phenix (*32*). Figures were prepared with ChimeraX (*33*).

### Mass spectrometry

The purified AMPA receptor complexes (∼1 μg of protein) from mouse sample were dried, dissolved in 5% SDS, 8 M urea, 100 mM glycine (pH 7.55), reduced with (tris(2-carboxyethyl)phosphine at 37°C for 15 min, alkylated with methyl methanethiosulfonate for 15 min at room temperature followed by addition of acidified 90% methanol and 100 mM triethylammonium bicarbonate buffer (TEAB; pH 7.55). The sample was then digested in an S-trap micro column briefly with 2 μg of a Tryp/LysC protease mixture, followed by a wash and 2 hours of digestion at 47°C with trypsin. The peptides were eluted with 50 mM TEAB and 50% acetonitrile, 0.2% formic acid, pooled, and dried. Each sample was dissolved in 20 μl of 5% formic acid and injected into Thermo Fisher Scientific QExactive HF mass spectrometer. Protein digests were separated using liquid chromatography with a Dionex Rapid Separation Liquid Chromatography (RSLC) ultra-high performance liquid chromatography (UHPLC) system and then delivered to a QExactive HF (Thermo Fisher Scientific) using electrospray ionization with an Easy Spray Source (Thermo Fisher Scientific). Xcalibur version 4.0 was used to control the system. Survey mass spectra were acquired over mass/charge ratio (*m/z*) 375 to 1400 at 120,000 resolution (*m/z* 200), and data-dependent acquisition selected the top 10 most abundant precursor ions for tandem mass spectrometry by higher-energy collisional dissociation (HCD) fragmentation using an isolation width of 1.2 *m/z*, normalized collision energy of 30, and a resolution of 30,000. Dynamic exclusion was set to auto, charge state for tandem mass spectrometry +2 to +7, maximum ion time of 100 ms, minimum automatic gain control (AGC) target of 3 × 10^6^ in MS1 mode and 5 × 10^3^ in MS2 mode.

Data analysis was performed using Comet (v. 2016.01, rev. 3) (*34*) against an October 2022 version of canonical FASTA protein database containing *Mus musculus* UniProt sequences and concatenated sequence-reversed entries to estimate error thresholds and 179 common contaminant sequences and their reversed forms. Comet searches for all samples performed with trypsin enzyme specificity with monoisotopic parent ion mass tolerance set to 1.25 Da and monoisotopic fragment ion mass tolerance set at 1.0005 Da. A static modification of +45.9877 Da was added to all cysteine residues and a variable modification of +15.9949 Da on methionine residues. A linear discriminant transformation was used to improve the identification sensitivity from the Comet analysis (*35*, *36*). Separate histograms were created for matches to forward sequences and for matches to reverse sequences for all peptides of seven amino acids or longer. The score histograms of reversed matches were used to estimate peptide false discovery rates (FDRs) and set score thresholds for each peptide class. The overall protein FDR was 1.2%.

### Western blot analysis

Purified AMPARs were separated by SDS-PAGE and transferred to nitrocellulose membranes. Primary antibodies were used at 1:1000 and included anti-GluA1 (Invitrogen, SD2010), anti-GluA2 (Thermo Fisher Scientific, PA5-19496), anti-GluA3 (Invitrogen, 32-0400), anti-GluA4 (Millipore, ab1508), anti-PSD95 (Abcam, ab-18258), and anti–TARP-γ2 (Addgene, N245/1R), as well as monoclonal antibodies against TARP-γ8 (13A8) and CNIH2 generated as described in (*5*). Membranes were incubated with IRDye 800 CW anti-mouse or anti-rabbit secondary antibodies at 1:10,000 for visualization.

### Animal use statement

For each native AMPAR preparation, five C57BL/6 mice (4 to 6 weeks, both male and female) were ordered from Charles River Laboratories. No randomization, blinding, or experimental manipulations were performed on these animals. All mice were euthanized under the Oregon Health & Science University (OHSU) Institutional Animal Care and Use Committee (IACUC) protocols, consistent with the recommendations of the Panel on Euthanasia of the American Veterinary Medical Association and carried out only by members of the E.G. laboratory approved under IACUC protocol TR03_IP00000905.

### Cell line statement

SF9 cells for the expression of baculovirus are from Thermo Fisher Scientific (12659017, lot 421973). The cells were not authenticated experimentally for these studies and tested negative for mycoplasma contamination.
