# Supplementary material for: Native AMPA receptor architecture reveals SynDIG4 engagement and auxiliary subunit heterogeneity
Source: Sci Adv. 2026 Jun 12;12(24):eaee7973. doi: 10.1126/sciadv.aee7973 (PMC13262616; doi:10.1126/sciadv.aee7973)
Supplement: Supplementary file 1 — Figs. S1 to S5 [file sciadv.aee7973_sm.pdf]

Science Advances
AAAS

Supplementary Materials for

Native AMPA receptor architecture reveals SynDIG4 engagement and auxiliary subunit heterogeneity

Chengli Fang and Eric Gouaux

Corresponding author: Eric Gouaux, gouauxe@ohsu.edu

Sci. Adv. **12**, eaee7973 (2026)
DOI: 10.1126/sciadv.aee7973

This PDF file includes:

Figs. S1 to S5

A

	Unique SpC	Coverage (%)		Unique SpC	Coverage (%)
GluA1	281	49	TARP γ7	6	58
GluA2	398	62	DLG4	46	49
GluA3	297	54	CPT1C	76	35
GluA4	73	42	ABHD6	49	45
TARP γ8	90	50	Shisa 9	38	32
TARP γ2	30	33	FRRS1L	27	39
TARP γ3	30	30	Noelin 1	25	40
TARP γ4	11	18	PRRT1	1	4

B

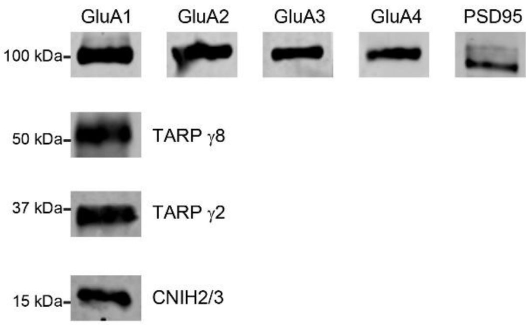

Fig. S1. Characterization of isolated GluA2-containing AMPARs. (**A**) Representative proteins of isolated native GluA2-containing AMPARs analyzed by mass spectrometry. SpC are spectral counts. (**B**) Western blot analysis of isolated AMPAR complexes using antibodies against GluA1, GluA2, GluA3, GluA4, PSD95, TARP-γ8, TARP-γ2 and CNIH2/3.

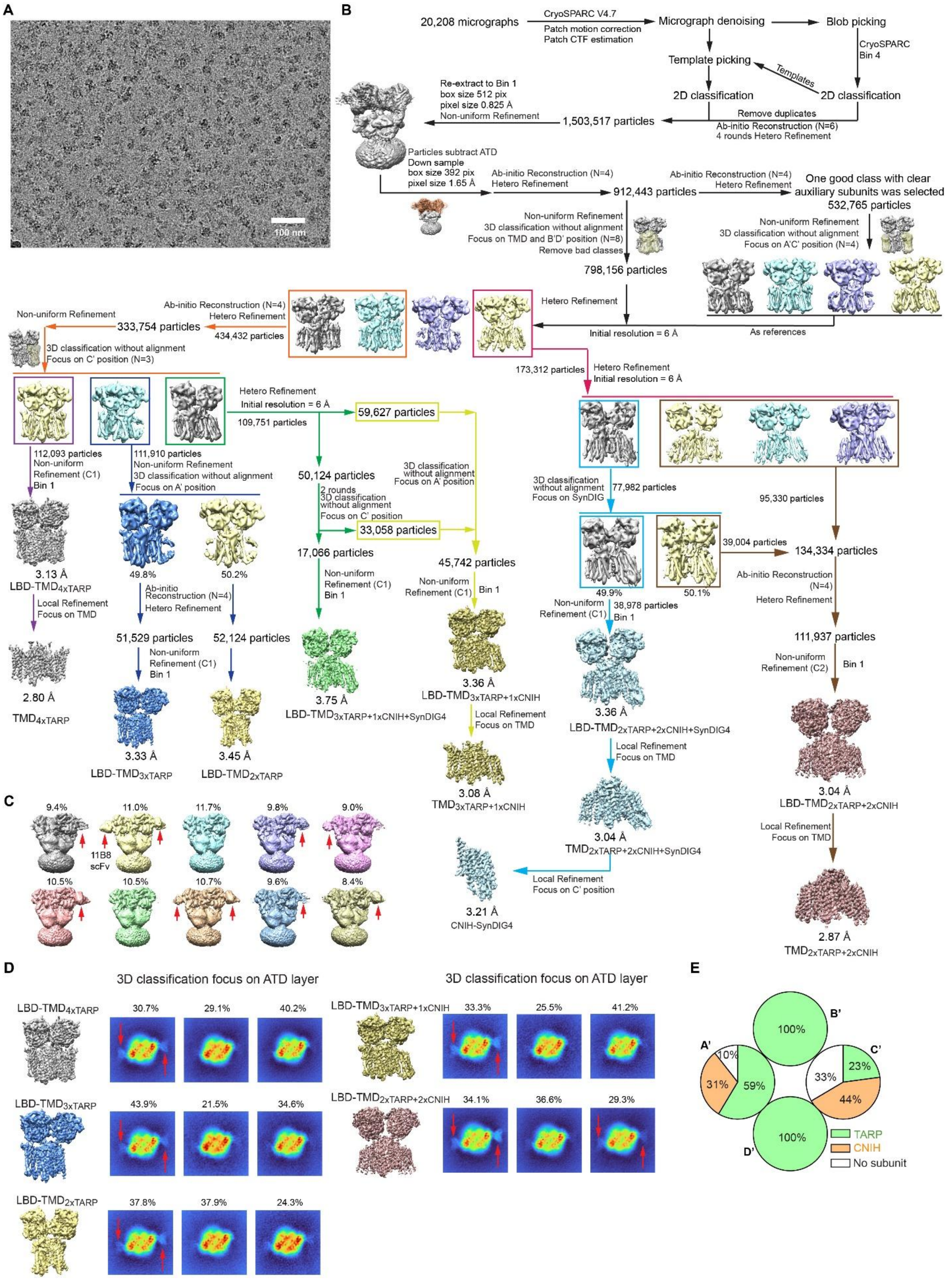
A
100 nm
B
20,208 micrographs
CryoSPARC V4.7
Patch motion correction
Patch CTF estimation
Micrograph denoising
Blob picking
CryoSPARC
Bin 4
Template picking
Templates
2D classification
2D classification
Re-extract to Bin 1
box size 512 pix
pixel size 0.825 Å
Non-uniform Refinement
1,503,517 particles
Remove duplicates
Ab-initio Reconstruction (N=6)
4 rounds Hetero Refinement
Particles subtract ATD
Down sample
box size 392 pix
pixel size 1.65 Å
Ab-initio Reconstruction (N=4)
Hetero Refinement
912,443 particles
Ab-initio Reconstruction (N=4)
Hetero Refinement
One good class with clear auxiliary subunits was selected
532,765 particles
Non-uniform Refinement
3D classification without alignment
Focus on TMD and B'D' position (N=8)
Remove bad classes
Non-uniform Refinement
3D classification without alignment
Focus on A'C' position (N=4)
798,156 particles
Hetero Refinement
Initial resolution = 6 Å
As references
Ab-initio Reconstruction (N=4)
Hetero Refinement
434,432 particles
Non-uniform Refinement
333,754 particles
3D classification without alignment
Focus on C' position (N=3)
173,312 particles
Hetero Refinement
Initial resolution = 6 Å
Hetero Refinement
Initial resolution = 6 Å
109,751 particles
59,627 particles
112,093 particles
Non-uniform Refinement (C1)
Bin 1
111,910 particles
Non-uniform Refinement
3D classification without alignment
Focus on A' position
50,124 particles
2 rounds
3D classification
without alignment
Focus on C' position
3D classification
without alignment
Focus on A' position
33,058 particles
17,066 particles
45,742 particles
3.13 Å
LBD-TMD4xTARP
49.8%
50.2%
Local Refinement
Focus on TMD
Ab-initio
Reconstruction (N=4)
Hetero Refinement
2.80 Å
TMD4xTARP
51,529 particles
52,124 particles
Non-uniform
Refinement (C1)
Bin 1
3.33 Å
LBD-TMD3xTARP
3.45 Å
LBD-TMD2xTARP
Non-uniform
Refinement (C1)
Bin 1
3.75 Å
LBD-TMD3xTARP+1xCNIH+SynDIG4
Non-uniform
Refinement (C1)
Bin 1
3.36 Å
LBD-TMD3xTARP+1xCNIH
Local Refinement
Focus on TMD
3.08 Å
TMD3xTARP+1xCNIH
3D classification
without alignment
Focus on SynDIG
77,982 particles
95,330 particles
49.9%
50.1%
39,004 particles
134,334 particles
Non-uniform
Refinement (C1)
38,978 particles
Bin 1
Ab-initio Reconstruction
(N=4)
Hetero Refinement
111,937 particles
Non-uniform
Refinement (C2)
Bin 1
3.36 Å
LBD-TMD2xTARP+2xCNIH+SynDIG4
Local Refinement
Focus on TMD
3.04 Å
TMD2xTARP+2xCNIH+SynDIG4
Local Refinement
Focus on C' position
3.21 Å
CNIH-SynDIG4
3.04 Å
LBD-TMD2xTARP+2xCNIH
Local Refinement
Focus on TMD
2.87 Å
TMD2xTARP+2xCNIH
C
9.4%
11.0%
11.7%
9.8%
9.0%
11B8
scFv
10.5%
10.5%
10.7%
9.6%
8.4%
D
3D classification focus on ATD layer
3D classification focus on ATD layer
LBD-TMD4xTARP
30.7%
29.1%
40.2%
LBD-TMD3xTARP
43.9%
21.5%
34.6%
LBD-TMD2xTARP
37.8%
37.9%
24.3%
LBD-TMD3xTARP+1xCNIH
33.3%
25.5%
41.2%
LBD-TMD2xTARP+2xCNIH
34.1%
36.6%
29.3%
E
B'
100%
A'
10%
31%
59%
C'
33%
23%
44%
D'
100%
TARP
CNIH
No subunit

Fig. S2. Cryo-EM data processing of native GluA2-containing AMPARs. (**A**) Representative motion-corrected micrograph. (**B**) Cryo-EM data processing workflow. (**C**) 3D classification focused on the ATD layer shows no additional densities at top of the receptor. Red arrows indicate the densities corresponding to the 11B8 scFv bound at the A′/C′ positions. (**D**) Focused 3D classification of the ATD layer for particles with different auxiliary subunit compositions reveals that auxiliary subunit assembly is independent of the specific AMPAR subunit arrangement. Based on previous reports (*9*) showing that GluA2 predominantly occupies the B and D positions, the class with two 11B8 scFv signals was defined as an A1A2A1A2 arrangement. Classes with one or absent 11B8 scFv signals may correspond to alternative arrangements such as A1A2AxA2 or AxA2AxA2. (**E**) Pie chart summarizing the distribution of auxiliary proteins around the receptor TMD, showing that the B′/D′ positions are fully occupied.

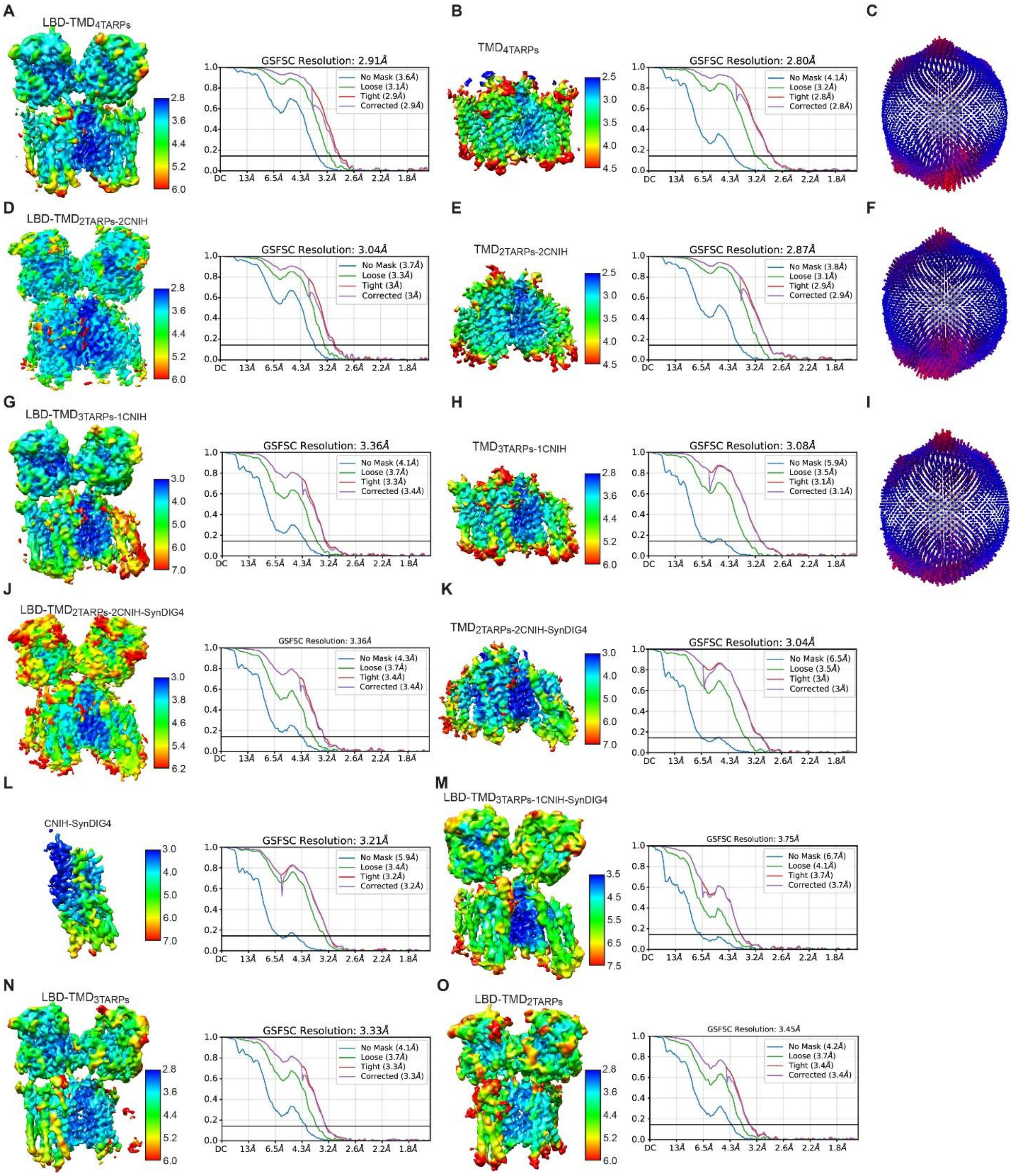

Fig. S3. Cryo-EM statistics of GluA2-containing AMPAR assemblies. (**A**) LBD–TMD map of the 4×TARP assembly. (**B**) TMD-focused map of the 4×TARP assembly. (**C**) Euler angle distribution of particles contributing to the 4×TARP reconstruction. (**D**) LBD–TMD map of the 2×TARP+2×CNIH assembly. (**E**) TMD-focused map of the 2×TARP+2×CNIH assembly. (**F**) Euler angle distribution of particles used for the 2×TARP+2×CNIH reconstruction. (**G**) LBD–TMD map of the 3×TARP+1×CNIH assembly. (**H**) TMD-focused map of the 3×TARP+1×CNIH assembly. (**I**) Euler angle distribution of particles used for the 3×TARP+1×CNIH reconstruction. (**J**) LBD–TMD map of the 2×TARP+2×CNIH+SynDIG4 assembly. (**K**) TMD-focused map of the

3×TARP+1×CNIH+SynDIG4 assembly. (**L**) Focused map of the SynDIG4–CNIH region. (**M**) LBD–TMD map of the 3×TARP+1×CNIH+SynDIG4 assembly. (**N**) LBD–TMD map of the 3×TARP assembly. (**O**) LBD–TMD map of the 2×TARP assembly. FSC curves are shown for each reconstruction, with the 0.143 criterion (black line) used to define resolution cutoffs. Local resolution estimates are colored according to the scale bar shown.

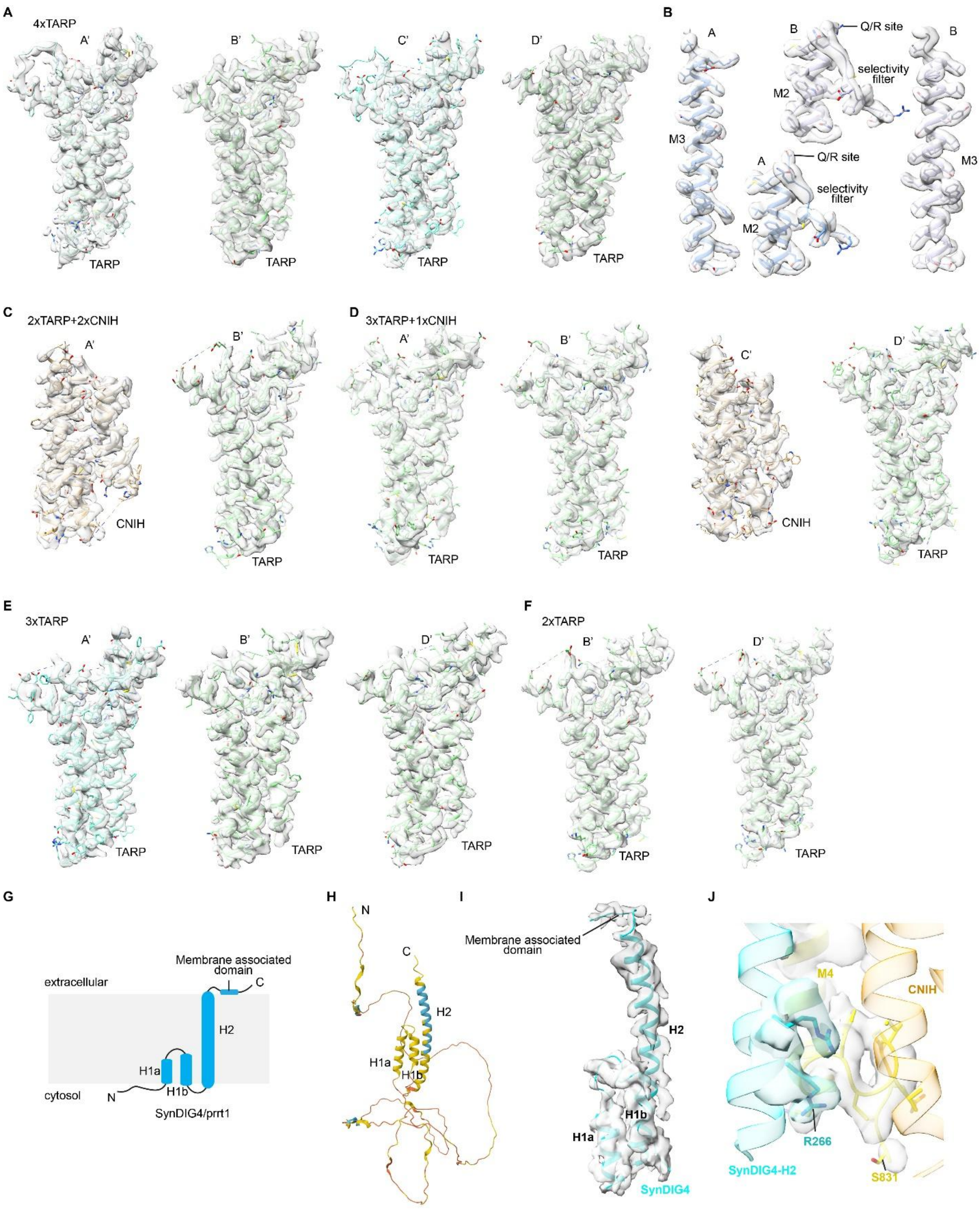

Fig. S4. Local density maps for reconstructions of GluA2-containing AMPAR assemblies. (**A**) Local density maps of the auxiliary subunit in the 4×TARP assembly. (**B**) The local maps show the M2 helices, the selectivity filters, and the M3 helices in the 4×TARP assembly. (**C**) Local density maps of the auxiliary subunit in the 2×TARP+2×CNIH assembly. (**D**) Local density

maps of the auxiliary subunit in the 3×TARP+1×CNIH assembly. (**E**) Local density maps of the auxiliary subunit in the 3×TARP assembly. (**F**) Local density maps of the auxiliary subunit in the 2×TARP assembly. (**G**) A cartoon showing the topology of SynDIG4/PRRT1. (**H**) The Alpha fold predicted structure model of SynDIG4/PRRT1. (**I**) Local map of SynDIG4/PRRT1 in 2×TARP+2×CNIH assembly showing clear density corresponding to the membrane-associated region, including one canonical transmembrane helix and two adjacent partial TMDs. (**J**) Map highlighting the proximity of SynDIG4 to the loop containing S831, suggesting a possible stabilizing effect on this region, relative to Fig. 2E.

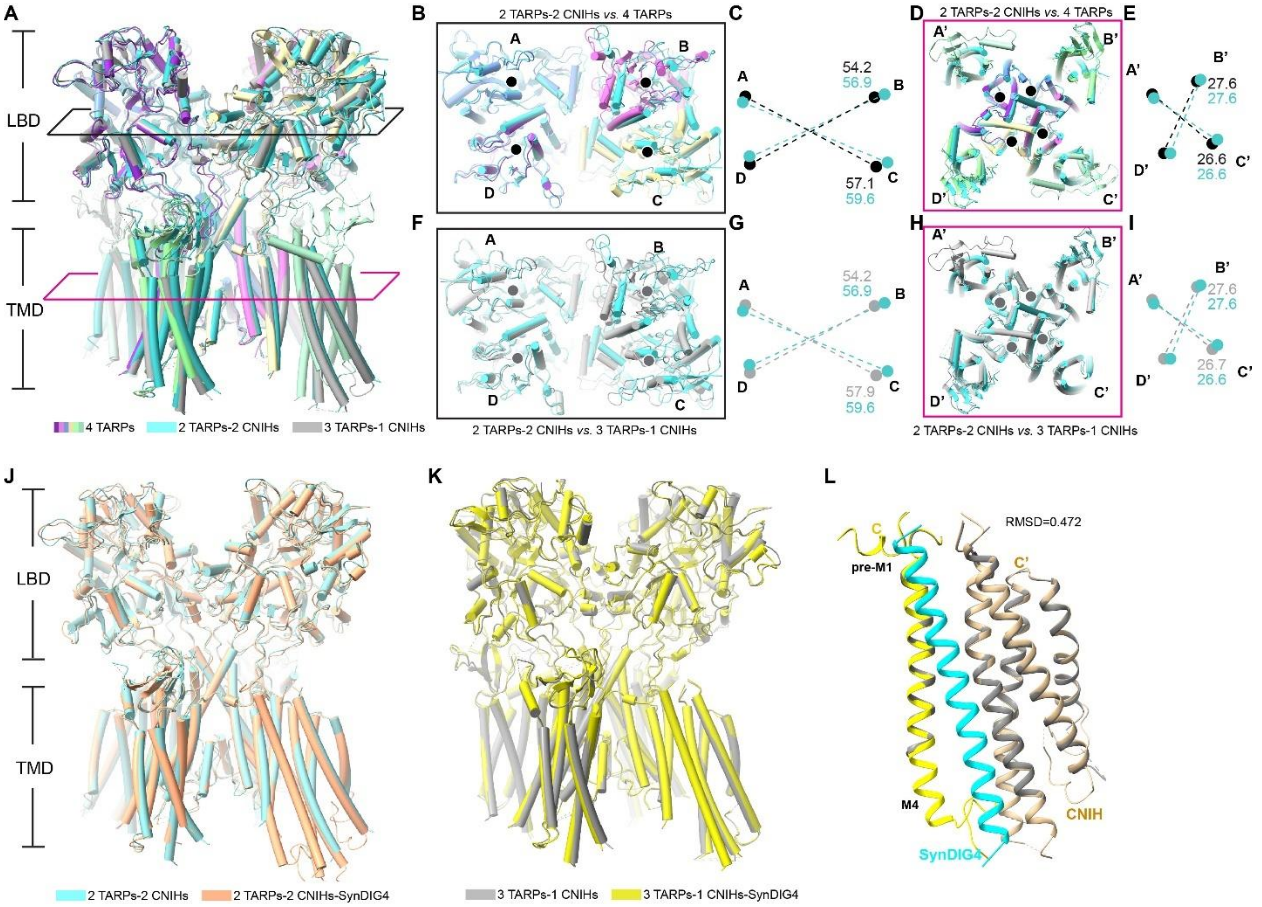

Fig. S5. Structural comparison of GluA2-containing AMPAR assemblies with fully occupied A′B′C′D′ positions. (**A-I**) Superposition of the LBD–TMD regions from the 4×TARP (colorful), 2×TARP+2×CNIH (cyan), and 3×TARP+1×CNIH (grey) assemblies. (B-E) Structural comparison of the LBD (B, C) and TMD (D, E) layers between the 4×TARP and 2×TARP+2×CNIH assemblies. (F-H) Structural comparison of the LBD (F, G) and TMD (H, I) layers between the 2×TARP+2×CNIH and 3×TARP+1×CNIH assemblies. Centers of mass (COMs) for the LBD and TMD layers of each subunit are indicated by colored circles. Schematic diagrams illustrate subunit arrangement differences, showing inter-subunit distances (in Å) in the LBD (C, G) and TMD (E, I) layers. (**J**) Superposition of LBD-TMD between 2×TARP+2×CNIH, and 2×TARP+2×CNIH+SynDIG4 assemblies. (**K**) Superposition of LBD–TMD between 3×TARP+1×CNIH and 3×TARP+1×CNIH+SynDIG4 assemblies. (**L**) Superposition of CNIH and M4 helix between 2×TARP+2×CNIH, and 2×TARP+2×CNIH+SynDIG4 assemblies. The 2×TARP+2×CNIH assembly is shown in gray, the 2×TARP+2×CNIH+SynDIG4 assembly is shown in color.
